# Evaluation of microhardness in two types of denture bases after using sodium hypochlorite and NatureDent disinfecting agents

**DOI:** 10.34172/joddd.2022.033

**Published:** 2022-11-15

**Authors:** Elnaz Moslehifard, Tahereh Ghaffari, Khosro Zarei, Mahsa Karimoghli

**Affiliations:** ^1^Department of Prosthodontics, Tabriz University of Medical Sciences, Tabriz, Iran; ^2^Department of Prosthodontics, Kurdistan University of Medical Sciences, Kurdistan, Iran

**Keywords:** Denture, Heat-cured acrylic resin, Microhardness, TiO2 nanoparticles

## Abstract

**Background.** Chemical agents, in combination with mechanical methods, play an important role in reducing microbial plaque on denture surfaces. However, these methods might change the mechanical behavior of acrylic resins, including microhardness and surface roughness. This in vitro study investigated the effect of two disinfectants, i.e., water and sodium hypochlorite, on the microhardness of conventional heat-cured and TiO_2_ nanoparticle-reinforced acrylic resins.

**Methods.** Sixty acrylic resin specimens were divided into two groups, and the samples in each group were randomly assigned to three subgroups (n=10). Heat-cured specimens and 1 wt% TiO_2_ acrylic resin were prepared and immersed in three solutions: water, a solution prepared with NatureDent pills, and 1% sodium hypochlorite for 30, 60, and 90 days. Microhardness tests were performed on each sample at each immersion stage. The data were analyzed using descriptive statistical methods, three-way and one-way ANOVA, repeated-measures *t* test, and Tukey HSD tests using SPSS 17. *P* values<0.05 were considered significant.

**Results.** All three independent parameters, including resin, solution, and time, significantly affected microhardness (*P*<0.05). The microhardness of both specimen types, i.e., conventional heat-cured and TiO_2_ nanoparticle-reinforced acrylic resins, immersed for 30, 60, and 90 days, was the highest and lowest in water and hypochlorite solutions, respectively. Regarding 90 days, the microhardness values of conventional heat-cured and TiO_2_ nanoparticle-reinforced acrylic resins were 17.050±0.094 and 19.953±0.053 in water, 15.675±0.069 and 18.965±0.037 in hypochlorite, and 16.713±0.122 and 19.39±20.113 in NatureDent solutions, respectively.

**Conclusion.** Disinfecting two types of acrylic resin specimens decreased their microhardness as a function of immersion time for up to 90 days in the three solutions. However, the magnitude of hardness lost was less for TiO_2_ nanoparticles-reinforced acrylic resin.

## Introduction

 Denture plaque is a significant factor in the etiology of opportunistic infections in elderly patients.^1^ Therefore, disinfection of dentures is recommended as a primary method for proper denture health. Different mechanical and chemical methods have been recommended for denture hygiene.^2^ Dental healthcare plays an essential role in maintaining oral mucosa health.^3^ However, these healthcare methods for the elderly are difficult due to illnesses or impaired skills; poor health care will lead to oral mucosa inflammation.^4^ Daily use of cleaning solutions has been recommended to prevent microbial colonization on the denture surface and promote oral hygiene. However, regular use of these solutions may affect the denture base resin’s physical and mechanical properties.^5^

 Characteristics commonly affected by denture cleansers are surface microhardness, denture roughness, and discoloration. These changes are critical to the long-term prognosis of any prosthesis.^1^ Surface microhardness of denture base resins is important because cleansing methods cause scratching and scrubbing of denture base surface; thus, sufficient resistance to these inevitable effects is necessary.^1,5^

 Denture cleaners are divided into different groups based on their chemical constituents, including alkaline peroxides, acids, enzymes, and alkaline hypochlorite.^6^ Previous studies have shown that repeated and prolonged use of disinfectants, such as sodium hypochlorite and sodium perborate, decreases the surface microhardness and microhardness of denture base resins.^5,7-9^

 Many studies have investigated the effect of disinfectants on the surface changes of acrylic resins. However, some considerations may necessitate further studies considering the variety of materials, acrylic resins, and immersion times.^10^

 Heat-cured acrylic resins are the most commonly used materials for fabricating dentures; however, they exhibit some inherent disadvantages, such as low flexural and impact strengths, necessitating methods to increase denture material strength, including polymethyl methacrylate/titanium dioxide. Shirkavandand Moslehifard^11^ explored the effect of incorporating TiO_2_ nanoparticles on the tensile strength of acrylic resins. They reported increased tensile strength of acrylic resin after incorporating 1% TiO_2_. However, increasing the nanoparticle percentage decreased the tensile strength. Also, Ahmed et al^12^ concluded that denture base microhardness significantly increased by adding 5% of TiO_2_, inversely affecting the denture base flexural strength.

 Previous studies suggested that denture base coating with TiO_2_ or adding TiO_2_ nanoparticles to acrylic resin could have antibacterial effects or inhibit biofilm formation on the denture surface.^13,14^

 Considering the discrepancies between different studies and the diversities involved in the use of various chemical compounds in cleansing denture bases by patients, as well as a paucity of evidence on TiO_2_-reinforced acrylic resin, the present study compared the effects of two chemical disinfectants on surface microhardness of conventional acrylic resins and heat-cured acrylic resins reinforced with 1 wt% TiO_2_ nanoparticles. The present study was conducted based on the hypothesis that “disinfection of conventional and TiO_2_-reinforced resins with chemical disinfectants influences the surface microhardness;” however, its extent was not clear.

## Methods

###  Preparation of samples 

 Sixty specimens were selected, with 30 made of conventional heat-cured acrylic resin (SR Triplex Hot, Germany, Liechtenstein, Ivoclar Vivadent) with dimensions of 20×20×20 mm^3^. They were mixed according to the manufacturer’s instructions and ISO 1567:1999 series for denture base polymers^15^ and pressed after molding. To cure the specimens, the flasks were immersed in a water bath and boiled for 45 minutes. Finally, the surface of the specimens was polished using sand papers (mesh #100, #200, and #400 grid silicon carbide STARCKE Papers, German Matador Brand) 10 times.

 Thirty specimens of TiO_2_-reinforced acrylic resin were made as follows: The first step was to mix conventional denture powder (polymethyl methacrylate) with 1 wt% of TiO_2 _particles under an ultrasonic device to obtain a homogeneous composition. They were molded, flasked, and cured according to the manufacturer’s instructions. To confirm the homogeneous distribution of nanoparticles, the specimens were examined by scanning electron microscopy (SEM) (Phenom, Model ProX, Eindhoven, the Netherlands) before the subsequent procedures.

###  Disinfection

 The samples were immersed in distilled water at 37°C for 48 ± 2 hours according to ADA and ISO standards.^15^ Two chemical disinfectants (1% sodium hypochlorite and NatureDent tablets) were used for immersion. The samples in each group were randomly assigned to three subgroups (n = 10) as follows:

1. The samples in subgroup 1 served as a control and were only kept in water.
2. The samples in subgroup 2 were kept in 1% sodium hypochlorite solution (containing 100 mL of sodium hypochlorite) (Iran, Robat Karim, Golrang, sodium hypochlorite) for 2 minutes with 30-, 60-, and 90-day cycles.^16^
3. The samples in subgroup 3 were kept in the sodium perborate disinfectant solution (NatureDent, Fittydent, Vienna, Austria) for 10 minutes with 30-, 60-, and 90-day cycles.

The immersion method was performed as follows: Each sample was immersed in a disinfecting solution for 2 or 10 minutes (depending on the disinfectant) at ambient temperature; then, each sample was retrieved and washed. In the next step, the samples were immersed in distilled water and transferred into a special container filled with distilled water due to assimilating storage conditions until the surface microhardness test was carried out. The distilled water of the container was exchanged between cycles of disinfection. The disinfecting solution was replaced daily, and the process was repeated daily. Microhardness tests were performed on one surface of samples at 30-, 60-, and 90-day intervals.

###  Evaluation of microhardness 

 The Vickers microhardness test was performed by SCTMC, model HV-1000Z, at Tabriz University Central Laboratory under an 0.49-N (50 g) force for 15 s. Each specimen was subjected to a hardness test three times with a force effect distance of 5 mm in each step, and the mean values of the three tests were reported.

###  Statistical analysis

 The data were analyzed using descriptive statistics (means, standard deviations, and frequency percentages). Three-way ANOVA was used to investigate the effect of three variables (acrylic resin type, disinfectant type, and time), and one-way ANOVA was used for effect analysis. Repeated-measures *t* test was used to analyze the effect of independent parameters during immersion time. Each variable was analyzed separately: *t* test for comparison of two types of acrylic resin and Tukey HSD test to classify variables using SPSS 17. In this study, a *P* value<0.05 was considered significant.

## Results

###  Scanning electron microscopy observation

**Figure 1:**
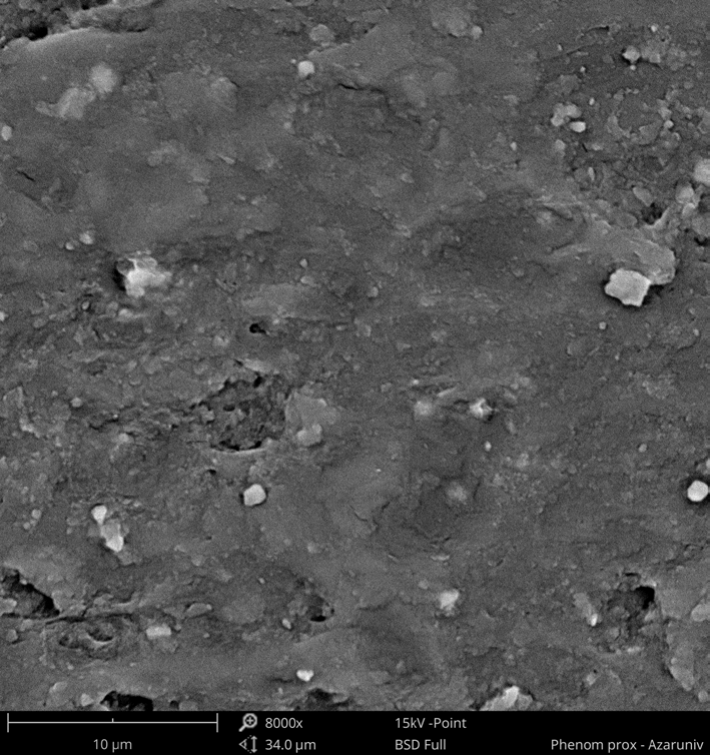
SEM image of the acrylic sample with 1% titanium dioxide (TiO_2_) particles distributed on the sample surface

Figure 1 shows a typical SEM image of acrylic resin with 1 wt% TiO_2_ nanoparticles. It can be seen that TiO_2 _nanoparticles are uniformly dispersed in the acrylic resin matrix.

###  Microhardness evaluation

Table 1 summarizes the results based on three-way ANOVA, which shows that all the three independent parameters, including resin, solution, and time significantly affected microhardness (*P*<0.05). Furthermore, the parameters were compared and summarized in Table 2 for the effect of time and solution. Table 3 shows the effect of time and solution, and Table 4 shows the effect of resin.

**Table 1:** Three-way ANOVA results for the effect of independent parameters of resin, solution, and time on microhardness

**Source**	**Type III sum of squares**	**df**	**Mean square**	**F**	***P***** value**
Resin	523.626	1	523.626	6.819E4	0.000
Solution	13.482	2	6.741	877.828	0.000
Time	9.516	3	3.172	413.081	0.000
Resin*solution*time	9.756	17	0.574	74.732	0.000
Error	1.659	216	0.008		
Total	80281.478	240			

*P *value: Three-way ANOVA.

**Table 2:** Comparison of microhardness values in different solutions in conventional acrylic resin

	**Conventional acrylic resin**						
	**Water**		**Sodium hypochlorite**		**NatureDent**		***P*** value*
	**Mean**	**SD**	**Mean**	**SD**	**Mean**	**SD**	
Day 1	17.05	0.094	17.024	0.074	17.041	0.123	0.838
Day 30	17.050^a^	0.094	16.566^c^	0.071	16.872^b^	0.123	0
Day 60	17.050^a^	0.094	16.112^c^	0.07	16.782^b^	0.123	0
Day 90	17.050^a^	0.094	15.675^c^	0.069	16.713^b^	0.122	0
*P* value	-		0.000		0.000		

*P *value: Repeated-measures (Sphericity Assumed and Greenhouse-Geisser). *P value: One-way ANOVA (Comparison of solutions). a,b,c: Tukey HSD test (similar letters represent non-significance).

**Table 3:** Comparison of microhardness of 1% TiO_2_-acrylic resin in different solutions at different time intervals

	**1% TiO**_2_						
	**Water**		**Sodium hypochlorite**		**NatureDent**		***P*** value*
	**Mean**	**SD**	**Mean**	**SD**	**Mean**	**SD**	
Day 1	19.953	0.053	19.97	0.041	19.921	0.115	0.363
Day 30	19.953^a^	0.053	19.700^b^	0.041	19.742^b^	0.113	0
Day 60	19.953^a^	0.053	19.371^c^	0.04	19.562^b^	0.113	0
Day 90	19.953^a^	0.053	18.965^c^	0.037	19.392^b^	0.113	0
*P*-value	-		0.000		0.000		

*P* value: Repeated-measures (Sphericity Assumed and Greenhouse-Geisser). *P value: One-way ANOVA (Comparison of solutions). a,b,c: Tukey HSD test (similar letters represent non-significance).

**Table 4:** Comparison of microhardness of two types of resin immersed in different solutions for four periods of times

		**Water**		**Sodium hypochlorite**		**NatureDent**	
		**Mean**	**SD**	**Mean**	**SD**	**Mean**	**SD**
Day 1	Conventional acrylic	17.05	0.094	17.024	0.074	17.041	0.123
	1% TiO_2_	19.953	0.053	19.97	0.041	19.921	0.115
	*P* value	<0.001		<0.001		<0.001	
Day 30	Conventional acrylic	17.05	0.094	16.566	0.071	16.872	0.123
	1% TiO_2_	19.953	0.053	19.7	0.041	19.742	0.113
	*P* value	<0.001		<0.001		<0.001	
Day 60	Conventional acrylic	17.05	0.094	16.112	0.07	16.782	0.123
	1% TiO_2_	19.953	0.053	19.371	0.04	19.562	0.113
	*P* value	<0.001		<0.001		<0.001	
Day 90	Conventional acrylic	17.05	0.094	15.675	0.069	16.713	0.122
	1% TiO_2_	19.953	0.053	18.965	0.037	19.392	0.113
	*P* value	<0.001		<0.001		<0.001	

*P* value: Independent t-test.

 Based on the repeated-measures t-test, the microhardness values of conventional acrylic resin were compared as a function of time, indicating that microhardness decreased in 90 days for both sodium hypochlorite and NatureDent solutions (*P*<0.05) (Table 2).

 A comparison of microhardness in different solutions based on one-way ANOVA showed no significant difference on the first day. However, on days 30, 60, and 90, the microhardness was maximum and minimum for water and sodium hypochlorite, respectively (*P*<0.05) (Table 2).

 Based on the repeated-measures t-test, microhardness values of 1% TiO_2_ acrylic resin significantly decreased during immersion for 90 days in both sodium hypochlorite and NatureDent solutions (*P*<0.05) (Table 3).

 Based on one-way ANOVA, a comparison of the microhardness in different solutions showed no significant difference after immersion for one day. However, on day 30, microhardness was the maximum for water and was the same for sodium hypochlorite and NatureDent solutions (*P*<0.05). On days 60 and 90, it was maximum in water but was minimum for sodium hypochlorite (*P*<0.05) (Table 3).

**Figure 2:**
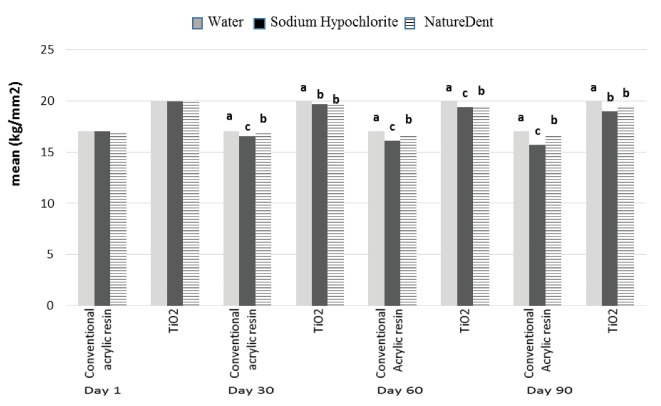
The comparison of the mean microhardness in two types of acrylic resin and three types of solution in the four-time intervals. Note: Tukey HSD test (similar letters represent non-significance)

 According to the data summarized in Table 4 and Figure 2, the microhardness of TiO_2_-reinforced acrylic resin was significantly higher than that of conventional resin in either sodium hypochlorite or NatureDent solution (*P*<0.05). This difference persisted at different time intervals.

## Discussion

 In this study, the research hypothesis was evaluated. A comparison of the groups indicated that the highest microhardness was related to the TiO_2_-reinforced acrylic resin samples, and the lowest microhardness was related to the conventional acrylic resin (*P* = 0.000).

 Daily use of disinfectant solutions can affect the physical and mechanical properties of denture base materials.

 Disinfectants, including water, vinegar, ethanol, alkaline peroxide, sodium hypochlorite, glutaraldehyde, and commercial disinfectant agents, have been used in different concentrations.^5,8,17-22^ Kurt et al^19^ evaluated the effect of cleaning solutions (alkaline peroxide, 1% sodium hypochlorite, and 0.1% polymeric-guanidine solution) on heat-polymerized PMMA and reported a decrease in the Vickers hardness, which was significantly higher in sodium hypochlorite. Similar results have been reported in studies^5,18,20,23^ where the hardness of heat-polymerized PMMAs decreased. Dayan^24^ reported that exposure to 5% sodium hypochlorite decreased the surface hardness of heat-polymerized PMMAs. In another study, 0.5% NaOCl solution effectively reduced microorganisms without significant changes in the color or roughness of the denture resin. The participants reported satisfaction with the cleaning results.^25^ De Freitas Fernandes et al^26^ and Daviet al^27^ found that sodium hypochlorite solution effectively removed all microorganisms at a low concentration of 1%. Neppelenbroek et al^5^ and Gornitskyet al^28^ showed that 1% sodium hypochlorite was a suitable cleanser to protect the denture base against microbial colonization and maintain oral health and denture hygiene. One percent sodium hypochlorite is a chlorine-based compound with hypochlorous acid, sodium, and water and is used as a disinfectant.^29^

 Ural et al^30^ found that for complete denture base cleaning, 10 minutes of daily immersion in sodium perborate cleaning solution is required. In the present study, NatureDent tablets, containing sodium perborate, was used every day for 10 minutes according to the manufacturer’s instructions.

 Disinfectants affected acrylic resin materials’ surface and mechanical characteristics over long periods. However, these effects have not been extensively studied after more extended immersion periods.^31,32^ Therefore, 30-, 60-, and 90-day time intervals were selected in this study to explore the effects of two disinfectants (1% sodium hypochlorite and NatureDent tablets) on the hardness of different acrylic resins.^33^

 This study showed that both disinfectants, especially 1% sodium hypochlorite, with 30, 60, and 90 days of immersion cycles decreased the microhardness of conventional heat-cured acrylic resin, corresponding to the periods, which might be attributed to continuous polymerization, the release of the residual monomers, and exposure of the monomers to oxygen free radicals.^7^

 Machado et al^1^ investigated the effect of repeated-immersion disinfection methods with sodium perborate and microwave on the hardness and roughness of heat-cured acrylic resin denture base (Lucitone 550) and two types of chairside relining resin (Kooliner and Duraliner II) for seven days. Disinfection by immersion in sodium perborate or microwave increased surface hardness in both relining resins, but the hardness of Lucitone 550 was not affected significantly with immersion or microwave. It should be noted that the study above did not consider long-term disinfection effects; therefore, it cannot be compared with our study.

 Moreno et al^34^ showed that after 12 days of disinfecting N1 resin (artificial sclera resin) and colorless ocular resin with neutral soap, Opti-free, Efferdent,1% hypochlorite, and 4% chlorhexidine, the greatest hardness changes were recorded in hypochlorite (similar to our study) and chlorhexidine groups.

 In a study by Goiato et al^8^ on four different types of acrylic resin (OndaCryl, QC 20, Classico, and Lucitone) and disinfection methods (microwave, Efferdent, 4% chlorhexidine, and 1% hypochlorite), the hardness decreased in all four types of acrylic resin after 60 days with thermal cycling and all methods of disinfection. However, these changes were within the clinically acceptable range.

 Neppelenbroek et al^5^ investigated three disinfectants (3.78% sodium perborate, 4% chlorhexidine gluconate, and 1% sodium hypochlorite) on two types of heat-cured acrylic resin (Lucitone 550 and QC-20) for 120 days, reporting that hardness decreased for up to 60 days, regardless of the type of acrylic resin and disinfection method similar to our research, but contrary to it, it remained stable after 60 days.

 Porwal et al^7^ studied the effects of disinfection with two common disinfectants, sodium perborate and sodium hypochlorite, on three denture base materials (conventional heat-cured resin, high-impact resin, and polyamide denture base resin), with a 180-day observation period. It was found that conventional heat-cured resin immersed in sodium perborate exhibited the greatest changes in hardness, which is contrary to our results, and all the samples exhibited a decrease in hardness after immersion in the disinfectant.

 In a study by Amin et al^24^ on conventional heat-cured acrylic resin for 60 days with 1% sodium hypochlorite and 2% alkaline glutaraldehyde, all the groups showed a decrease in hardness, with the highest decrease in alkaline glutaraldehyde.

 Pereira et al^35^ examined the microhardness of heat-cured acrylic resin after immersion in distilled water, domestic vinegar, 1% hypochlorite sodium, and hydrogen peroxide for 150 and 300 hours. The results showed no significant differences in the Knoop hardness between the groups. One of the reasons for the difference between these results and other studies is that the samples were continuously immersed in disinfectants for a short time (150 and 300 hours).

 Singh et al^36^ studied the effects of different disinfectants (distilled water, 0.5% sodium hypochlorite (NaOCl), and Daiso enzymatic cleanser) on the physical properties of different denture base materials (Trevalon PMMA, Valplast, and High Impact Trevalon). There was a significant difference in microhardness between the three denture base resins from baseline to six months. They concluded that considering the immersion duration, the concentration of the solution, the temperature of the solution, material properties, and its chemical composition are critical in the selection of denture cleansers. Most of the results reported above are consistent with our findings because by immersing acrylic resin in aqueous solutions, H_2_O molecules attach to the acrylic resin molecules through the adsorption mechanism. This binding causes expansion at the molecular level. This increase at the microscopic level is shown by the formation of bubbles on the surface. The presence of these absorption bumps can also affect the hardness because the more uneven the surface, the weaker the hardening effect is, and the microhardness values will be smaller. In other words, some of the indenter’s force is spent passing through the bubbles due to water absorption.

 In the present study, microhardness was significantly higher for 1% TiO_2_-reinforced acrylic resin than conventional acrylic resin after being disinfected in several different solutions.

 Several studies have shown that incorporating nanoparticles such as TiO_2, _nano-ZrO_2_, and aluminum borate whiskers can significantly improve acrylic resins’ flexural strength and surface hardness.^37^ Therefore, we focused on the effect of incorporating TiO_2 _nanoparticles into acrylic resins. In 2018, Aziz^38^ studied the effects of incorporating TiO_2_ nanoparticles on the properties of some high-impact acrylic resin using various processing techniques. A total of 120 high-impact acrylic resin samples were assigned to two groups based on curing methods (microwave or water bath), and each group was divided into two control subgroups (without TiO_2_) and a group with TiO_2 _nanoparticles. This study showed that the presence of TiO_2_ as a reinforcing substituent in dentures significantly increases the impact strength of acrylic resins. Furthermore, SEM images showed that the effect of TiO_2 _nanoparticles would be favorable when the particles are uniformly dispersed in an acrylic resin. In this regard, another study by Totu et al^14^ showed that increasing the concentration of TiO_2 _nanoparticles could lead to the formation of new groups and aggregates of particles instead of the homogeneous dispersion of TiO_2 _nanoparticles, affecting mechanical properties.

 Andreotti et al^39^ studied microhardness in N1 acrylic resin for artificial sclera by adding different nanoparticles (zinc oxide, TiO_2_, and barium sulfate with 1%, 2%, and 2.5% concentrations). The results showed that the microhardness of TiO_2_ and barium sulfate groups increased after the simulated aging process. Furthermore, after aging, significantly higher microhardness values were observed in the 1–2% TiO_2_ groups compared to other groups.

 Based on previous studies, adding a high amount of TiO_2_ nanoparticles adversely influences the impact and flexural strengths of acrylic resins.^40-42^ However, in a study by Ghahremani et al,^43^ the tensile and impact strengths of color-modified acrylic resin with 1% TiO_2 _were significantly higher than the conventional acrylic resin. Therefore, the effects of TiO_2 _nanoparticles on the mechanical properties of acrylic-based resins have been controversial and may depend on many parameters such as particles’ shape and size, synthesizing approaches of composite resins, etc.

 In another study, polymethyl methacrylate was reinforced with 0% (control), 2.5 wt%, and 5 wt% nanotubes of TiO_2_, increasing the hardness, flexural strength, and fracture toughness of PMMA.^44^ It seems methods of mixing and also the form of TiO_2_ particles affect mechanical parameters. TiO_2_ nanoparticles would influence the interaction of aqueous solutions used here, including water, sodium hypochlorite, and NatureDent. When TiO_2_ nanoparticles are added to the resins, they resist the penetration of water molecules into the structure, blocking the reaction sites between them. From the microhardness point of view, the presence of TiO_2 _as a hard ceramic material in the acrylic structure itself could be assumed as a hardener. In addition, the nanometer dimensions of TiO_2 _particles could lead to a further increase in hardness because it has been shown that the smaller the size of the ceramic particle in the polymer-ceramic composite, the stronger the combination. Moreover, strengthening would affect microhardness.^28,45^ Therefore, in the first place, the hardness of TiO_2_-reinforced acrylic resin is expected to be higher than conventional acrylic resin, consistent with this study.

 Further studies are necessary to investigate the effect of disinfection on conventional and reinforced resins. The use of other oxide particles, such as ZnO, can be of interest. The polymerization method of acrylic resins can be extended or modified using in-situ or solution-based processes to improve the distribution of particles in the resins. In addition, extending immersion times and bactericidal properties of the resins can be studied.

## Conclusion

 This study showed that the microhardness values of both conventional acrylic resin and 1% TiO_2_-reinforced acrylic resins were significantly higher in water compared with the other two aqueous solutions, i.e., hypochlorite and NatureDent. The microhardness of the specimens immersed in hypochlorite was the least. NatureDent solution led to microhardness values between those of the other solutions. Incorporating 1% titanium dioxide powder into acrylic resin could enhance the microhardness of denture bases. The effects of the two studied disinfectants on the microhardness were the same in both groups of acrylic resin and led to decreased microhardness over time.

 Clinicians may prefer NatureDent tablets instead of 1% sodium hypochlorite, for the disinfection of acrylic denture base materials to minimize plaque accumulation and maintain the microhardness of the denture base.

## Acknowledgments

 This article was written based on a dataset from an MSc thesis entitled “Investigating two types of denture disinfectant agents on microhardness and surface roughness of conventional heat-cured and titanium dioxide nanoparticle-reinforced acrylic resin” registered at Tabriz University of Medical Sciences, Faculty of Dentistry (reference number: 60415.T). The thesis was supported by the Vice Chancellor for Research at Tabriz University of Medical Sciences.

## Author Contributions

 EM initiated, conceptualized, and supervised the research work. SM, MG, and AF prepared samples and performed experiments with the collaboration of EM, TG, and FN. All authors have contributed to analyzing the data and writing the manuscript.

## Funding

 None.

## Ethics Approval

 This study was approved by the Ethics Committee of Tabriz University of Medical Sciences (No. IR.TBZMED.VCR.REC.1397.183)

## Competing Interests

 The authors declare that they have no conflicts of interest.
